# Do fathers have sufficient knowledge to administer medicine to children correctly?

**DOI:** 10.1093/eurpub/ckac130.042

**Published:** 2022-10-25

**Authors:** N Yanagi, H Satoh, Y Sawada

**Affiliations:** Graduate School of Pharmaceutical Sciences, University of Tokyo, Tokyo, Japan

## Abstract

**Background:**

Recently, the father's involvement in childcare is increasing in Japan. Inappropriate use of medication for children at home has been reported worldwide, however, the most responsible person was likely to be the mother. We aimed to compare the knowledge related to administering medication to children between fathers and mothers among Japanese parents.

**Methods:**

An online survey regarding medication administration to children was conducted in March 2022. Parents living with preschool children were recruited and categorized by four factors: sex (fathers and mothers), age of children, regular medication, and the difficulty level in giving medicine to their own children. The cross-sectional data were collected for each category. The knowledge related to administering medication to children was measured using ten statements such as “Children can be given a reduced dose of adult medicine” using a 5-point Likert scale. The answer “disagree” was defined as correct understanding and was compared between fathers and mothers (Chi-square test).

**Results:**

The participants were 145 fathers and 128 mothers. The percentage of fathers who answered all questions correctly or all questions incorrectly was 9.0% (mothers = 13.3%) and 25.5% (mothers = 13.3%) respectively. Each statement was answered correctly by fathers 20.0-57.9% and by mothers 25.8-71.9% and fathers were less likely to have the correct knowledge than mothers (6 items, p < 0.05). The biggest difference between those two groups was the statement of “Children should be given more than the proper dose for rapid effect”. Furthermore, the fathers having some difficulties in giving medicine had lower awareness about appropriate medication use than fathers having no difficulties.

**Conclusions:**

Fathers were more likely to have lower knowledge related to administering medication to children than mothers. Medical professionals like pharmacists will need to support fathers.

**Key messages:**

- Fathers were more likely to have lower knowledge related to administering medication to children than mothers among Japanese parents living with preschool children.
- To improve fathers’ knowledge related to administering medication to children, support by medical professionals like pharmacists will be needed.

